# Content analysis of policy documents related to non-communicable diseases prevention and control in Sri Lanka: a developing country in the South-East Asia

**DOI:** 10.12688/f1000research.144221.1

**Published:** 2024-03-11

**Authors:** Ishanka Talagala, Chrishantha Abeysena, Rajitha Wickremasinghe

**Affiliations:** 1 Department of Community Medicine and Family Medicine, Faculty of Medicine, University of Moratuwa, Moratuwa, Sri Lanka; 2 Post Graduate Institute of Indigenous Medicine, University of Colombo, Colombo, Sri Lanka; 3 Department of Public Health, Faculty of Medicine, University of Kelaniya, Ragama, Sri Lanka

**Keywords:** Non communicable diseases, policy, analysis, content, Sri Lanka, developing country, Asia, documents

## Abstract

**Background:**

Health policies form the foundation for provisioning best level care and are important for all stakeholders including patients and healthcare providers. Health policy analysis and evaluation allows policy makers to improve an existing policy, terminate a non-effective policy and to successfully implement future policies.

The objective was to assess the coherence between the two local policy documents on NCD prevention and control in Sri Lanka, the national NCD policy (NCD policy) and the multisectoral action plan (MSAP), and to assess the consistency of MSAP with the global action plan for NCDs.

**Methods:**

The content analysis of the NCD policy and MSAP of Sri Lanka was conducted based on the modified criteria developed to the ‘Analysis of determinants of policy impact’ model, by two reviewers independently. Coherence between MSAP and the global NCD action plan were also assessed by two reviewers independently. Consensus for discrepancy was achieved through discussion.

**Results:**

Accessibility was the strongest criteria for the NCD policy, while, resources and obligations were the weakest. Goals and monitoring and evaluation criteria were the strongest in the MSAP. Requirement for improvement were identified in policy background, goals, monitoring and evaluation, and public opportunities for the NCD policy. Accessibility, policy background, resources, public opportunities and obligations require further improvement in the MSAP. The MSAP is well coherent with the global road map for NCD prevention and control.

**Conclusion:**

Policy documents related to NCD prevention and control in Sri Lanka are coherent with the global action plan, while, there are areas within the local policy documents that need to be improved to enhance the coherence between the local documents. Lessons learnt by this activity need to be utilized by Sri Lanka and other countries to improve the uniformity between the NCD policy documents within the country as well as internationally.

**Abbreviations**
MSAP: Multisectoral action plan
NCD: Non communicable diseases
WHO: World health organization

## Introduction

Health policy is a plan, a statement of decisions, that is taken by those with responsibility to direct the investments and actions towards improving the health of the people, either by improving health care or by preventing diseases. It could manifest as a law, regulation, procedure, or guidelines ( Buse, Mays, & Walt, 2005). Health policy makers therefore have an important role to play in the process of health policy formulation, as they have the responsibility of developing a logical pathway to address a particular health issue considering the demand of the people and limited resources at hand. This is of utmost relevance and importance in low resource countries like Sri Lanka.

Health policy evaluation and analysis therefore allows the policy makers to improve an existing policy, terminate a non-effective policy and to successfully implement future policies. Despite its importance, however, policy analysis and evaluation in lower- and middle-income countries like Sri Lanka is not a common practice. In Sri Lanka, the state (through the Ministry of Health) is both the main provider and the purchaser of health services, and is also involved in policy making, regulating, research and training along with its administrative functions. Therefore, one of the issues that the Ministry of Health faces is the information asymmetry, as usually the relevant information is available only within a particular unit or an institution. As in many other developing countries, Sri Lanka has weak regulations, issues with poor documentation, low human and other resources, low capacity to monitor and evaluate, short-term nature of funds for policy research and dependence on external donor funds ( Walt *et al.*, 2008).

Nevertheless, assessing the internal validity of the policy documents is one way of improving the effective implementation of a health policy. Here, internal validity refers to the clarity and comprehensiveness of the policy statements within all the relevant policy documents, in order to achieve the expected outcomes of a particular policy. Analysis of the relevant policy documents, therefore, is a straightforward method to assess how they influence the successful implementation of the policy. In addition to internal validity of the local policy documents, it is important that a country’s policy documents are in line with international policies and global action plans. This would allow all the countries across the globe to implement certain actions collectively to combat a public health issue, allow for comparisons among the countries and share knowledge and resources.

Non communicable diseases (NCDs) account for 75% of all deaths while these diseases are among the top ten causes of deaths in Sri Lanka ( Senaratne & Mendis, 2018). 54% of NCD-related deaths among males and 36% of NCD-related deaths among females in 2015 were below 70 years of age ( Senaratne & Mendis, 2018). Pre-mature mortality and the chronic debilitating nature of these diseases requiring long term care and management, have major economic impacts on the individual, family and the country. In the Sri Lankan health system, where the bulk of the healthcare cost is borne by the state, this results in a major impact on the country’s economic development. Therefore, the development of effective NCD prevention policies and programmes is essential and worth investing in.

The national NCD prevention and control programme (administered by the ‘NCD unit’ and referred to hereafter in the document) was initiated in 2009; it was the first major attempt to address the spectrum of NCDs through documentation of the National policy and strategic framework for prevention and control of chronic non-communicable diseases (referred to as ‘NCD policy’ hereafter) ( Ministry of Health Sri Lanka, 2010). This policy addresses the prevention and control of cardiovascular diseases including coronary heart diseases, cerebrovascular diseases and hypertension; diabetes mellitus; chronic respiratory diseases and chronic renal diseases while other NCDs including cancers and mental illnesses are addressed through separate policy documents ( Ministry of Health Sri Lanka, 2010). The policy aims at addressing these diseases and their risk factors through primordial, primary, secondary and tertiary preventive strategies at different stages of the life cycle at different settings ( Ministry of Health Sri Lanka, 2010). Considering the important roles played by different stakeholders in the prevention and control of the NCDs, the Ministry of Health documented the national multisectoral action plan for the prevention and control of non-communicable diseases (MSAP) ( Ministry of Health Nutrition and Indigenous Medicine Sri Lanka, 2016), which iterates the responsibilities of, and activities for, each stakeholder to implement, to achieve the ultimate policy targets by the year 2025. Thus, the NCD unit of the Ministry of Health oversees the activities of stakeholders in order to achieve the NCD policy outcomes.

Based on the Sri Lankan STEPS survey data of 2021 ( Ministry of Health Sri Lanka, 2021), the overall current tobacco users has increased by 1.5%, with a 10.8% increase in current smokeless tobacco use, compared to 2015 ( Ministry of Health Nutrition and Indigenous Medicine Sri Lanka, 2015). Further, current alcohol consumers have increased by 15.6%; the proportion with insufficient physical activity has increased by 14.5%; the proportion who are overweight has increased by 34.5%; the proportion who are obese has increased by 86.4%; the proportion of hypertensives has increased by 33.3%; the proportion with raised fasting blood glucose has increased by 97.3% and the proportion with high total cholesterol level has increased by 106.4%, by 2021 ( Ministry of Health Sri Lanka, 2021) compared to 2015 ( Ministry of Health Nutrition and Indigenous Medicine Sri Lanka, 2015). However, current smokers have reduced by 6%; the proportion who consumed less than five servings of fruits and/or vegetables per day has reduced by 6.5%; the proportion who always or often added salt or salty sauces to their food before they eat or as they are eating has reduced by 83.9%; and the proportion of those who always or often consumed processed food high in salt has reduced by 69.2% compared to that of 2015 ( Ministry of Health Nutrition and Indigenous Medicine Sri Lanka, 2015).

Although, these data were collected in 2021, amidst the COVID-19 pandemic, it is important to note that these results were observed following the effective period of the NCD policy and the MSAP. Hence, as the first step in evaluating the NCD policy implementation, it is imperative to evaluate the NCD policy documents for coherence internally as well as internationally. This study, therefore, evaluated the policy documents related to the prevention and control of NCDs in Sri Lanka for their internal validity. Since it is important that the country’s activities to combat NCDs are in line with the global actions, the MSAP was also compared with the WHO global action plan for the prevention and control of NCDs 2013-2020 (referred to as ‘global action plan’ hereafter) ( World Health Organization, 2013), to assess its consistency with the global road map.

## Methods

Cheung *et al.* (2010) developed certain criteria to assess the alignment between policy statements and expected policy outcomes among policy documents related to the Australian chronic care programme. These criteria were based on the criteria validated by Rütten *et al.* (2003), which were initially based on von Wright’s concept of ‘logic of events’. This logic of events explains the factors that determine human action and the logic underlying the interaction between these factors. Thus, the model explains how the interaction between wants, abilities, duties and opportunities affect human behaviour, ultimately affecting the impact of a health policy. It explains that in certain circumstances with created opportunities for action through health policies, an individual would take actions based on their wants and duties, within their abilities. And all these actions would ultimately create new opportunities, resulting in further action ( Rütten *et al*., 2003).

Translating this logic of events into policy level, Rütten *et al.* (2011) developed and validated the ‘Analysis of determinants of policy impact (ADEPT) model’. In the ADEPT model, ‘wants’ gets translated in to ‘goals’; ‘duties’ to ‘obligations’; ‘abilities’ to ‘resources’; and ‘opportunities’ refers to the institutional/organizational, political and public opportunities, that would ultimately affect the impact of a health policy ( Rütten *et al.*, 2011). This model has been utilized in several policy analyses and development projects worldwide ( Rütten *et al.*, 2011). The logic of events model and the ADEPT model consider the interaction between the determinants of human behaviour and appreciate the fact that these relationships change with the context. This is similar to the influencing factors in the policy formulation process, which are ever changing as well.

Cheung *et al.* (2010) modified the criteria in the ADEPT model by adding 16 new criteria. Criteria with strong relevance to analysis of the policy context were excluded as it was not possible to assess the context through analysing the documents. Further details of the changes made to the original ADEPT model and the reasons are published elsewhere ( Cheung *et al.*, 2010).

**Figure 1.**
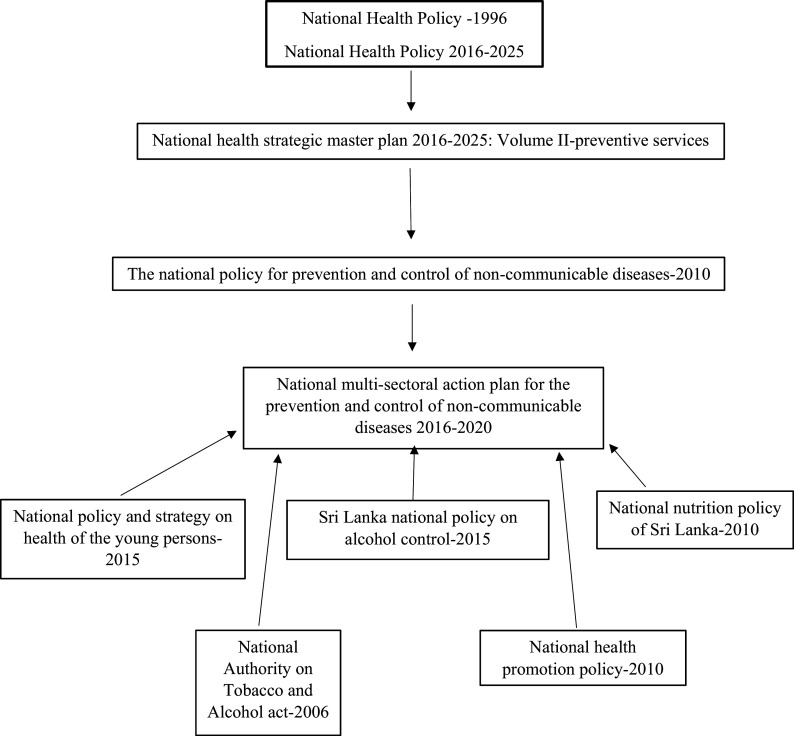
Map of policy documents related to the NCD prevention programme (Cardiovascular diseases, diabetes mellitus, chronic respiratory diseases and chronic renal diseases prevention and control programme).

The NCD prevention and control activities implemented in Sri Lanka are mainly based on the key strategic areas identified by the NCD policy ( Ministry of Health Sri Lanka, 2010). The implementation of these key strategies is through the activities identified in the MSAP ( Ministry of Health Nutrition and Indigenous Medicine Sri Lanka, 2016). However, at the implementation stage, other health policies such as the national nutrition policy, the national health promotion policy and the national authority on tobacco and alcohol (NATA) act govern the implementation as well. Figure 1 shows the map of the main policy documents related to the NCD prevention programme of Sri Lanka. However, since the national NCD policy and the MSAP are the policy documents that are directly considered for the prevention and control of NCDs in Sri Lanka, the analysis of the current study was limited to these two documents.

Analysis of the policy documents related to the prevention and control of NCDs in Sri Lanka was based on the modified criteria developed by Cheung *et al.* (2010), which are given in Table 1.

**Table 1.** Criteria for analysing policy documents based on Cheung *et al.* (2010).

Criteria for analysing policy documents
**A. Accessibility**
1. The policy document is accessible [hard copy and online].
**B. Policy Background (source of health policy)**
1. The scientific grounds of the policy are established. 2. The goals are drawn from a conclusive review of literature. 3. The source of the health policy is explicit. i. Authority [one or more persons, books scientific articles or sources of information]. ii. Quantitative or qualitative analysis. iii. Deduction [premises that have been established from authority, observation, intuition, or all three]. 4. The policy encompasses some set of feasible alternatives.
**C. Goals**
1. The goals are explicitly stated [the goals are officially spelled out]. 2. The goals are concrete enough [quantitative where possible and qualitative where not] to be evaluated later. 3. The goal is clear in its intent and in the mechanism with which to achieve the desired goals, yet does not attempt to prescribe in detail what the change must be. 4. The action centres on improving the health of the population. 5. The policy is supported by evidence of external consistency in logically drawing a health outcome from the goals and policy outcome. 6. The policy is supported by internal validity in logically drawing a health outcome from the goals and policy outcome.
**D. Resources**
1. Financial resources are addressed [there are sufficient financial resources] i. The cost of condition to community has been mentioned. ii. Estimated financial resources for implementation of the policy is given. iii. Allocated financial resources for implementation of the policy are clear. iv. There are reward/sanction for spending the allocated resources on other programmes. 2. Human resources are addressed [there is enough personnel]. 3. Organizational capacity is addressed [my organization has the necessary capacities].
**E. Monitoring and Evaluation**
1. The policy indicated monitoring and evaluation mechanisms. 2. The policy nominated a committee or independent body to perform the evaluation. 3. The outcome measures are identified for each of the explicit and implicit objectives. 4. The data, for evaluation, collected before, during and after the introduction of the new policy. 5. Follow up takes place after a sufficient period to allow the effects of policy change to become evident. 6. Other factors that could have produced the change [other than policy] identified. 7. Criteria for evaluation are adequate and clear.
**F. Political Opportunities** **
1. Co-operation between political levels involved [federal, state, area health] has either worsened or improved. ** 2. Support from other sectors [economy, science, justice] has either worsened or improved. ** 3. The political climate has either worsened or improved. ** 4. Cooperation between public and private organizations has either worsened improved. ** 5. The lobby for the action has either worsened or improved. **
**G. Public Opportunities**
1. The media’s interest has either worsened or improved. ** 2. The population supports the action. ** 3. Multiple stakeholders are involved. 4. Primary concerns of stakeholders recognised and acknowledged to obtain long term support. 5. There is media’s interest. **
**H. Obligations**
1. The obligations of the various implementers are specified-who has to do what? 2. The action is part of health professionals’ existing duties. ** 3. Scientific results are compelling for action. 4. Health professional obliged to the population to act in this area. **

** Excluded criteria from the current analysis as these items could not be directly assessed by assessing only the policy documents.

Criteria A to H except criterion F, were considered as ‘fulfilled/strong’ if all the criteria were addressed within the policy document; if only some criteria were addressed, it was considered as ‘room for improvement’ and if none of the criteria were addressed, it was considered as ‘not fulfilled/weak’ ( Cheung *et al*., 2010).

Two expert reviewers independently analysed the national NCD policy and the MSAP using the criteria given in Table 1, in 2020, prior to the COVID-19 pandemic in the country. Any discrepancy between the two reviewers for any of the criteria were further discussed to arrive at a consensus, and any further disagreement was resolved through an assessment by a third independent reviewer.

Furthermore, the MSAP was compared with the global action plan for combating NCDs, for consistency between the two policy documents, by the two expert reviewers independently as well. Any discrepancy between the two reviewers for any of the criteria were further discussed to arrive at a consensus.

## Results

Tables 2a and 2b include review of the NCD policy and the MSAP based on the criteria given in Table 1.

**Table 2a.** Content analysis of the NCD policy and multisectoral action plan - Sri Lanka.

Criteria	NCD Policy-2009			Multi-sectoral action plan 2016-2020		
	Fulfilled or strong	Room for improvement	Not fulfilled or weak	Fulfilled or strong	Room for improvement	Not fulfilled or weak
**A. Accessibility**						
1. The policy document is accessible (hard copy and online)	Y			Y		
**B. Policy Background (source of health policy)**						
1. The scientific grounds of the policy are established	Y			Y		
2. The goals are drawn from a conclusive review of literature		Y			Y	
3. The source of the health policy is explicit						
i. Authority (one or more persons, books scientific articles or sources of information)	Y			Y		
ii. Quantitative or qualitative analysis	Y			Y		
iii. Deduction (premises that have been established from authority, observation, intuition, or all three)		Y			Y	
4. The policy encompasses some set of feasible alternatives			Y			Y
**C. Goals**						
1. The goals are explicitly stated (The goals are officially spelled out)	Y			Y		
2. The goals are concrete enough (quantitative where possible and qualitative where not) to be evaluated later	Y			Y		
3. The goal is clear in its intent and in the mechanism with which to achieve the desired goals, yet does not attempt to prescribe in detail what the change must be		Y		Y		
4. The action centres on improving the health of the population	Y			Y		
5. The policy is supported by evidence of external consistency in logically drawing a health outcome from the goals and policy outcome		Y			Y	
6. The policy is supported by internal validity in logically drawing a health outcome from the goals and policy outcome		Y		Y		
**D. Resources**						
1. Financial resources are addressed [there are sufficient financial resources]			Y		Y	
i. The cost of condition to community has been mentioned			Y		Y	
ii. Estimated financial resources for implementation of the policy is given			Y	Y		
iii. Allocated financial resources for implementation of the policy are clear			Y		Y	
iv. There are reward/sanction for spending the allocated resources on other programmes			Y			Y
2. Human resources are addressed [there is enough personnel]			Y		Y	
3. Organizational capacity is addressed [my organization has the necessary capacities]			Y			Y
**E. Monitoring and Evaluation**						
1. The policy indicated monitoring and evaluation mechanisms	Y			Y		
2. The policy nominated a committee or independent body to perform the evaluation		Y		Y		
3. The outcome measures are identified for each of the explicit and implicit objectives			Y	Y		
4. The data, for evaluation, collected before, during and after the introduction of the new policy			Y	Y		
5. Follow up takes place after a sufficient period to allow the effects of policy change to become evident			Y	Y		
6. Other factors that could have produced the change (other than policy) identified			Y			Y
7. Criteria for evaluation are adequate and clear			Y	Y		
**G. Public opportunities**						
1. Multiple stakeholders are involved		Y		Y		
2. Primary concerns of stakeholders recognised and acknowledged to obtain long term support			Y			Y
**H. Obligations**						
1. The obligations of the various implementers are specified-who has to do what?			Y	Y		
2. Scientific results are compelling for action		Y			Y	

Y = Yes.

**Table 2b.** Overall opinion of the reviewers.

Criteria	NCD policy-2009			Multisectoral action plan 2016-2025		
	Fulfilled or strong	Room for improvement	Not fulfilled or weak	Fulfilled or strong	Room for improvement	Not fulfilled or weak
A. Accessibility	Y				Y	
B. Policy background		Y			Y	
C. Goals		Y		Y		
D. Resources			Y		Y	
E. Monitoring and evaluation		Y		Y		
G. Public opportunities		Y			Y	
H. Obligations			Y		Y	

Y = Yes.

*Accessibility:* the two core policy documents for prevention and control for NCDs are currently accessible on the internet, the Ministry of Health website and the unit website. And the NCD unit web site is linked to the Ministry of Health website as well. Nevertheless, the accessibility and the availability of these documents to the field health staff, who are the implementers of the NCD policy and the action plan, is unclear and could not be assessed in this study.

*Policy background:* The NCD policy and the MSAP was written with authority, using statistics and deduction to provide the scientific background for the policy. The literature review included peer reviewed journal articles, reports of the World Health Organization, Ministry of Health Sri Lanka, World Bank, and information from the Registrar General’s Department.

*Goals:* Goals in both the NCD policy and the MSAP were explicit, concrete and were qualitative. The actions are centred on improving the health of the population. The goal of the MSAP is more or less similar to the goal of the global action plan for the prevention and control of NCDs: 2013-2020 ( World Health Organization, 2013) ( Table 3), giving more emphasis to multisectoral collaboration while, the goal of the NCD policy is stated as “To reduce the burden due to chronic NCDs by promoting healthy lifestyles, reducing the prevalence of common risk factors, and providing integrated evidence-based treatment options for diagnosed NCD patients” ( Ministry of Health Sri Lanka, 2010). The NCD policy objective states the quantitative policy goal of a 2% annual reduction of premature mortality due to NCDs through implementation of the key strategies to combat NCDs in the country ( Ministry of Health Sri Lanka, 2010).

**Table 3.** Comparison between the Global Action plan and MSAP, Sri Lanka.

Components of the Global action plan	Components in the National Multi-sectoral Action Plan of Sri Lanka	Similarity
**Vision:** A world free of the avoidable burden of non-communicable diseases	**Vision:** A country free of the avoidable burden of non-communicable diseases	Yes
**Goal:** To reduce the preventable and avoidable burden of morbidity, mortality and disability due to non-communicable diseases by means of multisectoral collaboration and cooperation at national, regional and global levels, so that populations reach the highest attainable standards of health and productivity at every age those diseases are no longer a barrier to well-being or socioeconomic development	**Goal:** To reduce the preventable and avoidable burden of morbidity, mortality and disability due to non-communicable diseases by means of multisectoral collaboration and cooperation at national, level, so that populations reach the highest attainable standards of health and productivity at every age those diseases are no longer a barrier to well-being or socioeconomic development	Yes
**Overarching principles:** Life course approach; empowerment of people and communities; evidence based strategies; universal health coverage; management of real, perceived or potential conflicts of interest; human rights approach; equity-based approach; national action and international cooperation and solidarity; multisectoral action	**Overarching principles:** Life course approach; empowerment of people and communities; evidence based strategies; universal health coverage; management of real, perceived or potential conflicts of interest; human rights approach; equity-based approach; national action and international cooperation and solidarity; multisectoral action	Yes
**Nine (09) voluntary global targets** 1. A 25% relative reduction in premature mortality from cardiovascular disease, cancer, diabetes or chronic respiratory diseases 2. At least 10% relative reduction in the harmful use of alcohol, as appropriate, within the national context 3. A 10% relative reduction in prevalence of insufficient physical activity 4. A 30% relative reduction in mean population intake of salt/sodium 5. A 30% relative reduction in prevalence of current tobacco use in persons aged 15+ years 6. A 25% relative reduction in prevalence of raised blood pressure and or contain the prevalence of raised blood pressure, according to national circumstances 7. Halt the rise in diabetes and obesity 8. At least 50% of eligible people receive drug therapy and counselling (including glycaemic control) to prevent heart attacks and strokes 9. An 80% availability of the affordable basic technologies and essential medicines, including generics, required to treat major non-communicable diseases in both public and private facilities	**Nine (09) voluntary global targets** 1. A 25% relative reduction in premature mortality from cardiovascular disease, cancer, diabetes or chronic respiratory diseases 2. A 10% relative reduction in the use of alcohol 3. A 10% relative reduction in prevalence of insufficient physical activity 4. A 30% relative reduction in mean population intake of salt/sodium 5. A 30% relative reduction in prevalence of current tobacco use in persons aged over 15 years 6. A 25% relative reduction in prevalence of raised blood pressure and or contain the prevalence of raised blood pressure 7. Halt the rise in obesity and diabetes 8. A 50% of eligible people receive drug therapy and counselling (including glycaemic control) to prevent heart attacks and stroke 9. An 80% availability of affordable basic technologies and essential medicines including generics, required to treat major non-communicable diseases in both public and private facilities	Yes
**Menu of policy options given in the Global action plan**	**Main activity areas identified in the National Multi-sectoral Action Plan of Sri Lanka**	**Similarity**
**Objective 1: To raise the priority accorded to the prevention and control of noncommunicable diseases in global, regional and national agendas and internationally agreed development goals, through strengthened international cooperation and advocacy**	**Objective 1: To raise the priority accorded to the prevention and control of noncommunicable diseases in global, regional and national agendas and internationally agreed development goals, through strengthened international cooperation and advocacy**	Yes
Raise public and political awareness, understanding and practice about prevention and control of NCDs	• Establish A National NCD council and convene regular meetings bi annually • Develop advocacy packages on prevention and control of NCDs for politicians, each level in the health sector and non- health sectors at national, provincial and district levels • Advocacy meetings for the upper-level managers of the relevant ministries, authorities and departments at the national, provincial and district levels • Advocacy meeting for the political authorities • Design and launch a public education campaign on NCD	Yes
Integrate NCDs into the social and development agenda and poverty alleviation strategies	• Integrate the prevention and control of NCDs in to national planning processes and broader development agendas • Share process indicators and global NCD targets with the national team working on Sustainable Development Goals	Yes
Strengthen international cooperation for resource mobilization, capacity-building, health workforce training and exchange of information on lessons learnt and best practices	• Conduct advocacy meetings with the officers in the Ministry of Finance and UN agencies for adequate funding • Initiate action to obtain the required human resources for NCD related work • Capacity building of relevant staff on NCD Prevention and Control	Yes
Engage and mobilize civil society and the private sector as appropriate and strengthen international cooperation to support implementation of the action plan at global, regional and national levels	• Conduct workshops at National, provincial and district levels to map stake holders and to design health promotion activities • Establish provincial and district level multi sectoral committees and monitor implementation of existing policies	Yes
**Objective 2: To strengthen national capacity, leadership, governance, multisectoral action and partnerships to accelerate country response for the prevention and control of noncommunicable diseases**	**Objective 2: To strengthen national capacity, leadership, governance, multisectoral action and partnerships to accelerate country response for the prevention and control of noncommunicable diseases**	Yes
Prioritize and increase, as needed, budgetary allocations for prevention and control of NCDs without prejudice to the sovereign right of nations to determine taxation and other policies	• Conduct advocacy meetings with the officers in the Ministry of Finance and UN agencies	Yes
Assess national capacity for prevention and control of NCDs	• Ensure activities relevant to different units of the MoH are coordinated to strengthen the linkages between different units of Ministry of Health for NCD prevention and control	Yes
Develop and implement a national multisectoral policy and plan for the prevention of control of NCDs through multi-stakeholder engagement	• Already available	Yes
Strengthen national capacity including human and institutional capacity, leadership, governance, multisectoral action and partnerships for prevention and control of noncommunicable diseases	• Initiate action to obtain the required human resources for NCD related work • Capacity building of relevant staff on NCD Prevention and Control	Yes
**Objective 3: To reduce modifiable risk factors for noncommunicable diseases and underlying social determinants through creation of health-promoting environments**	**Objective 3: To reduce modifiable risk factors for noncommunicable diseases and underlying social determinants through creation of health-promoting environments**	Yes
**Tobacco Use**	**Tobacco Use**	Yes
Implement WHO Framework Convention on Tobacco Control (FCTC) • Reduce affordability of tobacco products by increasing tobacco excise taxes • Create by law completely smoke-free environments in all indoor workplaces, public places and public transport • Warn people of the dangers of tobacco and tobacco smoke through effective health warnings and mass media campaigns • Ban all forms of tobacco advertising, promotion and sponsorship	• Accelerate full implementation of FCTC • Bring about amendments to NATA Act 2006 • Advocate to raise tobacco tax • Setup tobacco cessation services • Strengthen the services available for tobacco cessation • Improve community awareness on tobacco use including the use of smokeless tobacco • Conduct trade seminars in view of establishing tobacco free zones • Conduct awareness programmes for media personnel • Conduct tobacco research • Strengthen NATA • Train the health staff on tobacco (including smokeless) prevention and control	Yes
**Harmful Use of Alcohol**	**Harmful Use of Alcohol**	Yes
Implement the WHO global strategy to reduce harmful use of alcohol through multisectoral actions in the recommended target areas	• Implement the National Alcohol Policy	Yes
Strengthening awareness of alcohol-attributable burden; leadership and political commitment to reduce the harmful use of alcohol	• Monitor prevalence of alcohol among adults and harmful effects of alcohol in the country and share the findings • Reduce alcohol related violence and injuries	Yes
Providing prevention and treatment interventions for those at risk of or affected by alcohol use disorders and associated conditions	• Establish treatment and rehabilitation services related to alcohol	Yes
Supporting communities in adopting effective approaches and interventions to prevent and reduce the harmful use of alcohol	• Establish a mechanism to implement, monitor and evaluate alcohol policy at national and district levels	Yes
Implementing effective drink–driving policies and countermeasures	• Implement the National Alcohol Policy • Establish a mechanism to implement, monitor and evaluate alcohol policy at national and district levels	Yes
Regulating commercial and public availability of alcohol	• Implement the National Alcohol Policy • Establish a mechanism to implement, monitor and evaluate alcohol policy at national and district levels	Yes
Restricting or banning alcohol advertising and promotions	• Implement the National Alcohol Policy • Establish a mechanism to implement, monitor and evaluate alcohol policy at national and district levels	Yes
Using pricing policies such as excise tax increases on alcoholic beverages	• Implement the National Alcohol Policy • Establish a mechanism to implement, monitor and evaluate alcohol policy at national and district levels	Yes
Reducing the negative consequences of drinking and alcohol intoxication, including by regulating the drinking context and providing consumer information	• Reduce alcohol related violence and injuries • Monitor prevalence of alcohol among adults and harmful effects of alcohol in the country and share the findings	Yes
Reducing the public health impact of illicit alcohol and informally produced alcohol by implementing efficient control and enforcement systems	• Reduce production and sale of illicit alcohol • Monitor prevalence of alcohol among adults and harmful effects of alcohol in the country and share the findings	Yes
Developing sustainable national monitoring and surveillance systems using indicators, definitions and data collection procedures compatible with WHO’s global and regional information systems on alcohol and health	• Conduct STEP survey every 4-5yrs	Yes
**Unhealthy diet & physical inactivity**	**Unhealthy diet & physical inactivity**	Yes
Implement the WHO Global Strategy on Diet, Physical Activity and Health	• Accelerate the implementation of the diet component of the Global Strategy on Diet, Physical Activity and Health	Yes
Increase consumption of fruit and vegetables	• Increase availability of fruits and vegetables	Yes
To provide more convenient, safe and health-oriented environments for physical activity	• Conduct advocacy meetings for town planners, politicians to improve environmental changes to promote physical activity • Establish/Strengthen the PA programmes in work places • Strengthen the PA programmes in the schools • Improve facilities for physical activity for the community • Develop physical activity guidelines for public	Yes
Implement WHO recommendations on the marketing of foods and non-alcoholic beverages to children	• Introduce food labeling to indicate unhealthy foods • Increase availability of food based dietary guidelines • Make available data and initiate policies to increase intake of healthy foods	Yes
Implement the WHO global strategy for infant and young child feeding	• Promote maternal and child nutrition	Yes
Reduce salt intake	• Develop and implement a national salt reduction strategy	Yes
Replace trans fats with unsaturated fats	• Take measures to reduce trans-fat in processed foods	Yes
Implement public awareness programmes on diet and physical activity	• Improve awareness of the public on cardio metabolic risk of consuming unhealthy foods through a mass media campaign • Conduct awareness programmes on healthy foods and food based dietary guidelines • capacity building of health workers on food based dietary guidelines	Yes
Manage food taxes and subsidies to promote healthy diet	• Increase tax for unhealthy food	Yes
**Objective 4: To strengthen and orient health systems to address the prevention and control of noncommunicable diseases and the underlying social determinants through people-centered primary health care and universal health coverage**	**Objective 4: To strengthen and orient health systems to address the prevention and control of noncommunicable diseases and the underlying social determinants through people-centered primary health care and universal health coverage**	Yes
Integrate very cost-effective noncommunicable disease interventions into the basic primary health care package with referral systems to all levels of care to advance the universal health coverage agenda	• Increase availability and access to NCD screening services • Improve NCD management at Primary Health Care level • Improve access to services to manage cardiovascular diseases • Improve access to services to manage Diabetes Mellitus • Improve access to services to screen Chronic Respiratory Diseases (CRD) - Asthma and chronic obstructive pulmonary disease (COPD) • Improve access to services to manage CRD • Improve Cancers screening services	Yes
Explore viable health financing mechanisms and innovative economic tools supported by evidence	• Develop policies for sustainable health financing for NCD	Yes
Scale up early detection and coverage, prioritizing very cost-effective high-impact interventions including cost-effective interventions to address behavioral risk factors	• Increase availability and access to NCD screening services • Publicize screening services through a mass media campaign	Yes
Train the health workforce and strengthen capacity of health system particularly at primary care level to address the prevention and control of noncommunicable diseases	• Capacity building of healthcare workers to manage NCDs • Establish a Council to produce clinical guidelines on NCDs	Yes
Improve the availability of the affordable basic technologies and essential medicines, including generics, required to treat major noncommunicable diseases, in both public and private facilities	• improve availability and access to essential NCD medicine and technologies • Improve the diagnostic and treatment facilities for cancer	Yes
Strengthen and orient health systems to address noncommunicable diseases and risk factors through people-centred primary health care and universal health coverage	• Establish health promotion settings • Improve NCD management at Primary Health Care level	Yes
Develop and implement a palliative care policy using cost-effective treatment modalities, including opioids analgesics for pain relief and training health workers	• Improve availability and access to palliative care • Improve palliative care for cancer patients	Yes
**Objective 5: To promote and support national capacity for high-quality research and development for the prevention and control of noncommunicable diseases**	**Objective 5: To promote and support national capacity for high-quality research and development for the prevention and control of noncommunicable diseases**	Yes
Develop and implement a prioritized national research agenda for noncommunicable diseases	• Establish a national multi-disciplinary research committee • Prepare a national research agenda for NCD • Establish a forum/committee to translate research in to policy action	Yes
Prioritize budgetary allocation for research on noncommunicable disease prevention and control	Conduct advocacy meetings with the officers in the Ministry of Finance and UN agencies	Yes
Strengthen human resources and institutional capacity for research	• Capacity building of officers on research	Yes
Strengthen research capacity through cooperation with foreign and domestic research institutes	• Identify research questions to support the implementation, monitoring and evaluation of the NCD action plan • Conduct priority research	Yes
Promote and support national capacity for high-quality research, development and innovation	• Conduct priority research • Capacity building of officers on research	Yes
**Objective 6: To monitor the trends and determinants of noncommunicable diseases and evaluate progress in their prevention and control**	**Objective 6: To monitor the trends and determinants of noncommunicable diseases and evaluate progress in their prevention and control**	Yes
Develop national targets and indicators based on global monitoring framework and linked with a multisectoral policy and plan	• Develop a monitoring framework to assess progress towards the goal • Conduct periodic NCD programme evaluation • Monitoring and evaluation framework for health system intervention	Yes
Strengthen human resources and institutional capacity for surveillance and monitoring and evaluation	• Improve staff and resources dedicated for data management in NCD	Yes
Establish and/or strengthen a comprehensive noncommunicable disease surveillance system, including reliable registration of deaths by cause, cancer registration, periodic data collection on risk factors and monitoring national response	• Improve staff and resources dedicated for data management in NCD	Yes
Integrate noncommunicable disease surveillance and monitoring into national health information systems	• Integrate NCD monitoring into HMIS	Yes
Monitor trends and determinants of noncommunicable diseases and evaluate progress in their prevention and control	• Conduct national review meetings • Conduct district review meetings	Yes

*Resources:* Financial resources for the implementation of the NCD policy is included as a strategic objective in the NCD policy and is worded as “Ensure sustainable financing mechanisms that support cost-effective health interventions at both preventive and curative sectors” ( Ministry of Health Sri Lanka, 2010), even though the allocated amount for the implementation of the policy, or the financial resources are not mentioned. The cost to the community due to NCDs is not explicitly mentioned either. Moreover, human resources for the implementation of the NCD policy is also addressed as a strategic objective within the policy as “Enhance human resource development to facilitate NCD prevention and care” ( Ministry of Health Sri Lanka, 2010).

The estimated financial resources for the implementation of the MSAP has been estimated as LKR 15.2 billion (approximately USD 46,704,563 currently, based on the conversion rate of 1 USD = LKR 325.45 on 23 ^rd^ October 2023) at its launch in 2016 ( Ministry of Health Nutrition and Indigenous Medicine Sri Lanka, 2016). The MSAP identifies the sources of funding including a loan provided by the World Bank for health system improvement, and a proposed loan from JICA ( Ministry of Health Nutrition and Indigenous Medicine Sri Lanka, 2016). As in the policy document, human resources and the organizational capacity were not addressed in the MSAP possibly due to specific activities not being identified to achieve the strategic objectives within the NCD policy.

*Monitoring and evaluation:* In the NCD policy, the coordination mechanisms are clearly articulated with the onus being on the NCD unit under the Director NCD, the focal point in the Ministry of Health for policy implementation, monitoring and evaluation. The outcome measures, data required and criteria for evaluation, however, are not explicitly stated. A results-based monitoring and evaluation system was to be established to evaluate the implementation ( Ministry of Health Sri Lanka, 2010). This gap is due to the lack of concrete goals and activities identified within the policy to achieve the ultimate policy objective. The policy does not mention about periodical follow up and monitoring to assess the effects of the policy.

On the other hand, the MSAP included a framework for monitoring progress in implementation based on inputs, processes and outcomes. Based on this framework, outcomes were to be assessed in the short-term (in 2018), the medium-term (in 2020) and in the long-term (in 2025). Although not explicitly stated, it appears that assessment of outcomes would be done by the NCD unit under the Director NCD of the Ministry of Health.

*Public opportunities:* The NCD policy and the National Health Council acknowledge stakeholder involvement and promote inter-sectoral and inter-ministerial collaboration. The National NCD steering committee has representation from Secretaries of relevant Ministries including that of Finance, Trade, Agriculture, Urban Planning, Education, Justice, Poverty Alleviation, and Social Welfare, and secretaries and directors of Provincial Health Ministries ( Ministry of Health Sri Lanka, 2010). In the action plan, multiple stakeholders are engaged in preventive activities, and it is specified as who should do what and when. However, both the documents have failed to identify the primary concerns of the stakeholders.

*Obligations:* The obligations of the various implementers are clearly stated in the MSAP. However, granting of any rewards or penalties related to fulfilment of these obligations is unclear.

Then, a comparison was made for congruence of the MSAP and the Global action plan for NCDs ( Table 3).

While the vision and goal of the MSAP were more or less similar to the global action plan ( World Health Organization, 2013), the overarching principles, the nine voluntary global targets and the objectives of MSAP were the same as that of the global action plan ( World Health Organization, 2013). The policy options iterated in the global action plan were then compared with the main activity areas identified in the national MSAP of Sri Lanka. All the policy options provided for countries in the WHO global action plan were found to be addressed via several activity areas in the MSAP of Sri Lanka ( Table 3).

## Discussion

We assessed the content and the comprehensiveness of the two main policy documents related to NCD prevention and control in Sri Lanka. These documents included the national policy and strategic framework for prevention and control of chronic non-communicable diseases - 2010 and the national multi-sectoral action plan for prevention and control of non-communicable diseases 2016-2020. Many of the evaluation criteria listed in Table 1 were met by the NCD policy and MSAP documents. Accessibility was the strongest for the NCD policy, while resources and obligations were the weakest. Goals and monitoring and evaluation criteria were the strongest in the MSAP. However, opportunities for improvement were identified in the policy background, goals, monitoring and evaluation, and public opportunities for the NCD policy. Accessibility, policy background, resources, public opportunities and obligations require further improvement in the MSAP. The comparison of the content of the MSAP and the WHO Global action plan for the prevention and control of non-communicable diseases 2013-2020 revealed that the vision, goal, overarching principles, the nine voluntary global targets and the objectives were more or less similar. Furthermore, there were similarities between the policy options identified in the global action plan and the various activities identified in the MSAP, indicating that the MSAP of Sri Lanka is in line with the global action plan as well.

Mapping of the policy documents related to NCD prevention and control enabled us to understand which documents are directly related with the NCD prevention and control and which are overlapping at the stage of implementation of the policy. The criteria developed by Cheung *et al.* (2010) enabled us to review and understand the comprehensiveness of the policy documents for NCD prevention and control in Sri Lanka. The criteria allowed a comprehensive content analysis of the policy documents and pinpoint the areas that require improvement. A comprehensive and clearly articulated policy document would increase the use of the policy document by the implementers as it clearly specifies the scientific background, and the different roles and responsibilities of stakeholders and implementers. This will directly increase the successful implementation of the policy as well.

Since the national programmes are guided by the policy document and is implemented through the multi-sectoral action plan, it is essential that these documents are widely disseminated and easily accessible to all the stakeholders, especially to the programme implementers. It is also important that the dissemination mechanism be adapted to the facilities and ground realities of different countries. In Sri Lanka, while, the NCD policy has been effective from 2010-2020, it was reported that by 2012, only 24.2% in the country were computer literate, with only 20.6% having access to the internet (within and outside household) ( Department of Census and Statistics Sri Lanka, 2012). Therefore, the availability and accessibility of policy documents by means other than through internet should have been promoted. However, these statistics have improved following the COVID-19 pandemic. Nevertheless, only 44.5% household population were using internet by the year 2021, with low usage in rural and estate sectors of the country ( Department of Census and Statistics, 2021). Also, having the majority of health staff (perhaps doctors being the exception) and the population being technologically challenged with only 34.3% being computer literate and only 57.2% being digitally literate in the country in 2021 (again, with low computer and digital literacy in rural and estate sectors of the country) ( Department of Census and Statistics, 2021), it is imperative that the policy documents be disseminated and made available and accessible through other mechanisms. However, given the background of limited printing due to unavailability of relevant resources in the current economic crises, it is essential that novel means need to be thought of in disseminating the policy documents.

Clear goal setting would strengthen a policy document as its use becomes easier for implementers, stakeholders and the public. Thus, setting clear goals to be achieved under each strategic objective of the NCD policy would increase the utilization of the policy and increase its successful implementation; in addition, it will make it easier for implementers, and internal and external evaluators to monitor and evaluate the implementation and the impacts of the policy.

The financial and non-financial resources required for implementation of the policy and the action plan should be explicitly stated in the documents. The estimated values and the action plan to obtain these resources along with a situational analysis on the currently available resources are important for the implementers of the policy. Documenting the current situation would allow easy evaluation of whether the required resources have been obtained or not during policy implementation. It would also allow policy implementers to prioritize activities, especially in low resource settings like in Sri Lanka. In addition, this will improve the understanding of the context within which the policy is implemented.

Documentation of the penalties for using allocated funds for any other activity would prevent disorganized use of funds based on personal or political opinion or influence, increasing the likelihood of successful implementation of the policy by ensuring the required flow of funds. This is important in low resource settings like Sri Lanka, with competing interests. Nevertheless, considering Sri Lanka, it is important to draw one’s attention towards the political and economic contexts within which the NCD policy and the MSAP have being effective. The country faced a separatist war till 2009, the global recession in 2009 largely affected developing countries like Sri Lanka and probably for many years to come ( Koh & Yu, 2020), the slowing of the world economy since mid-2018 ( Koh & Yu, 2020), the COVID-19 pandemic and the current economic crisis ( Ahmed, 2023; World Bank, 2021) in the country resulted in diversion of funds and therefore has adversely affected the optimal implementation of the NCD policy. Hence, the impact of COVID-19 and the impact of the economic crises on the health system and other systems in the country need to be well considered in the future, when implementing and assessing public health policies including the NCD policy.

Documenting the monitoring and evaluation framework and the establishment of an independent body for evaluation is essential in all policy documents to ensure accountability of policy implementation and evaluation.

Chronic NCDs has now become a pandemic affecting all countries across the globe with a major impact on developing nations ( World Health Organization, 2022). Therefore, WHO member countries collectively agreed upon the global action plan for the prevention and control of NCDs at the 66 ^th^ World Health Assembly, provisioning a road map for the stakeholders in order to combat NCDs ( World Health Organization, 2013). Therefore, while coherence between the local policy documents is essential for effective implementation of activities countrywide to combat NCDs, it is of important that the local policy documents are coherent with international policies and guidelines as well. Such consistency would allow easy international comparison of actions undertaken to combat NCDs between countries to understand the strategies that are effective in the global challenge to overcome NCDs.

## Conclusion and recommendations

While the policy documents related to NCD prevention and control in Sri Lanka are in coherence with the global action plan for the prevention and control of NCDs, there are many areas within these policy documents that need to be improved, to improve the coherence between the local documents. Lessons learned from this activity should be utilized to improve the future policy documentation in Sri Lanka. Further, lessons learnt from this activity may be utilized by other countries across the globe as well to improve the consistency between the NCD policy documents within the country as well as internationally.

## Data Availability

The data for this article consists of bibliographic references, which are included in the References section.
