# Supplementary material for: Content analysis of policy documents related to non-communicable diseases prevention and control in Sri Lanka: a developing country in the South-East Asia
Source: F1000Res. 2024 Mar 11;13:171. [Version 1] doi: 10.12688/f1000research.144221.1 (PMC11358681; doi:10.12688/f1000research.144221.1)
Supplement: Peer review history

# Reviewer report: Reviewer response for version 1

**DOI:** 10.5256/f1000research.157980.r313917

**Published:** 2024-08-28

**Authors:** Kalpana Kosalram

**Affiliations:** 1 SRM Institute of Science and Technology (Deemed to be University), Kattankulathur, Tamil Nadu, India

The Title and Abstract are detailed to make clear to the readers what the research is about. However, in the Abstract, the first line under ‘Results’ mentions the term "Accessibility" identified as the strongest criteria for the NCD policy, can mean accessibility of the policy itself, accessibility of healthcare services, or something else to the readers.  Please expand to make this clearer.

 The Introduction offers a well-justified explanation within the context of the available evidence. The discussion and concluding statements of the manuscript provide valuable pointers for uptake by other policymakers in low- and middle-income countries (LMICs) and could be further corroborated with information presented in the Introduction. The second paragraph of the Introduction briefly addresses the shortcomings in health policy evaluation and analysis in LMICs, with a more detailed focus on Sri Lanka. To strengthen the Introduction, it would be beneficial to include a few lines explaining why policy analysis is often overlooked in LMICs and how this gap negatively affects health outcomes.

 The methodology applied is replicable. The strength of this research work is the use of the ADEPT model and the criteria developed by Cheung et al. (2010) to assess the internal validity of policy documents.

 Although the mapping and analysis was limited to the national NCD policy and the MSAP, the results section presents a thorough review of these two policy documents, highlighting areas of strength and weakness. Being a content analysis of policy documents, this study does not require explicit ethical considerations. However, addressing potential biases in document analysis or discussing the ethical implications of health policy decisions could have added depth to the analysis.

 The discussion appropriately interprets the findings. The conclusion under Abstract and under Conclusion section mentions to "enhance the coherence between the local documents." The authors could rephrase or include the word ‘internal coherence’ to make the opening statement appear slightly less contradictory.  

 Overall, this study provides a valuable contribution to the field of health policy analysis, particularly in the context of NCD prevention and control in Sri Lanka. The Discussion and Conclusion section could expand offering more specific recommendations from the lessons learnt from this policy mapping activity, such as (i) incorporating other data sources for a more comprehensive understanding of the policy’s effectiveness like interviews or case studies (ii) proposing alternative dissemination methods (iii) proposing strategies for improving the internal coherence between local documents.

Is the work clearly and accurately presented and does it cite the current literature?

Yes

If applicable, is the statistical analysis and its interpretation appropriate?

Not applicable

Are all the source data underlying the results available to ensure full reproducibility?

Yes

Is the study design appropriate and is the work technically sound?

Yes

Are the conclusions drawn adequately supported by the results?

Yes

Are sufficient details of methods and analysis provided to allow replication by others?

Yes

Reviewer Expertise:

Health Management, Public Health Monitoring and Evaluation, Project Management

I confirm that I have read this submission and believe that I have an appropriate level of expertise to confirm that it is of an acceptable scientific standard.

# Reviewer report: Reviewer response for version 1

**DOI:** 10.5256/f1000research.157980.r291931

**Published:** 2024-08-01

**Authors:** Emanuel Orozco-Núñez

**Affiliations:** 1 National Institute of Public Health, Cuernavaca, Morelos, Mexico

Dear authors,

 Congratulations for your well written paper. I found your arguments about the content of the NCD policy in Pakistan very clear and coherent. It is relevant the way in which you integrate your theoretical framework and your methods; these procedures provide consistency to your work. Nevertheless that you analyzed only two documents, the procedure is rigorous. 

 One situation that results problematic is to think NCD combat as a bunch of good intentions. In this sense, your paper provides evidence about the relevance of strengthening health systems and provide enough financing. It is noticeable that in LMICs some health improvement objectives look like wishful thinking without the sufficient financial support. Your paper provides arguments for questioning this practical situation.

 Perhaps your conclusions can be more strong if you specify what specific areas of the policy instruments should be improved; otherwise, argument looks vague. The same with the lessons learned, please specify what. 

 With kind regards,

Is the work clearly and accurately presented and does it cite the current literature?

Yes

If applicable, is the statistical analysis and its interpretation appropriate?

Not applicable

Are all the source data underlying the results available to ensure full reproducibility?

Yes

Is the study design appropriate and is the work technically sound?

Yes

Are the conclusions drawn adequately supported by the results?

Partly

Are sufficient details of methods and analysis provided to allow replication by others?

Yes

Reviewer Expertise:

Health policy and Systems research.

I confirm that I have read this submission and believe that I have an appropriate level of expertise to confirm that it is of an acceptable scientific standard.
